# Bibliometric Assessment of European and Sub-Saharan African Research Output on Poverty-Related and Neglected Infectious Diseases from 2003 to 2011

**DOI:** 10.1371/journal.pntd.0003997

**Published:** 2015-08-11

**Authors:** J. Gabrielle Breugelmans, Michael M. Makanga, Ana Lúcia V. Cardoso, Sophie B. Mathewson, Bethan R. Sheridan-Jones, Karen A. Gurney, Charles S. Mgone

**Affiliations:** 1 European & Developing Countries Clinical Trials Partnership (EDCTP), The Hague, The Netherlands; 2 Evidence, Thomson Reuters, Intellectual Property & Science, Leeds, United Kingdom; The George Washington University School of Medicine and Health Sciences, UNITED STATES

## Abstract

**Background:**

The European & Developing Countries Clinical Trials Partnership (EDCTP) is a partnership of European and sub-Saharan African countries that aims to accelerate the development of medical interventions against poverty-related diseases (PRDs). A bibliometric analysis was conducted to 1) measure research output from European and African researchers on PRDs, 2) describe collaboration patterns, and 3) assess the citation impact of clinical research funded by EDCTP.

**Methodology/Principal Findings:**

Disease-specific research publications were identified in Thomson Reuters Web of Science using search terms in titles, abstracts and keywords. Publication data, including citation counts, were extracted for 2003–2011. Analyses including output, share of global papers, normalised citation impact (NCI), and geographical distribution are presented. Data are presented as five-year moving averages. European EDCTP member countries accounted for ~33% of global research output in PRDs and sub-Saharan African countries for ~10% (2007–2011). Both regions contributed more to the global research output in malaria (43.4% and 22.2%, respectively). The overall number of PRD papers from sub-Saharan Africa increased markedly (>47%) since 2003, particularly for HIV/AIDS (102%) and tuberculosis (TB) (81%), and principally involving Southern and East Africa. For 2007–2011, European and sub-Saharan African research collaboration on PRDs was highly cited compared with the world average (NCI in brackets): HIV/AIDS 1.62 (NCI: 1.16), TB 2.11 (NCI: 1.06), malaria 1.81 (NCI: 1.22), and neglected infectious diseases 1.34 (NCI: 0.97). The NCI of EDCTP-funded papers for 2003–2011 was exceptionally high for HIV/AIDS (3.24), TB (4.08) and HIV/TB co-infection (5.10) compared with global research benchmarks (1.14, 1.05 and 1.35, respectively).

**Conclusions:**

The volume and citation impact of papers from sub-Saharan Africa has increased since 2003, as has collaborative research between Europe and sub-Saharan Africa. >90% of publications from EDCTP-funded research were published in high-impact journals and are highly cited. These findings corroborate the benefit of collaborative research on PRDs.

## Author Summary

The European & Developing Countries Clinical Trials Partnership (EDCTP) was created in 2003 as a European response to the global health crisis caused by the three main poverty-related diseases (PRDs) of HIV/AIDS, tuberculosis and malaria. EDCTP funds research focusing on clinical trials for diagnosing, preventing and treating these diseases. We conducted a bibliometric analysis to 1) measure research output and citation impact from European and African researchers working on PRDs, 2) describe collaboration patterns, and 3) assess the citation impact of research funded by EDCTP. Citation analysis is a commonly used bibliometric tool to analyse scientific literature. Overall, the volume and citation impact of papers from sub-Saharan Africa has increased since 2003, as has collaborative research between Europe and sub-Saharan Africa. Papers arising from collaborative research had a higher citation impact than non-collaborative research and >90% of publications from EDCTP-funded research projects were published in high-impact journals. These results suggest that research on PRDs in sub-Saharan Africa is growing and that the EDCTP partnership contributes to high-impact, collaborative research published in high-impact journals. By providing research funds and supporting activities to strengthen the research environment, the partnership contributes to sub-Saharan African researchers taking the lead in PRD research.

## Introduction

The European & Developing Countries Clinical Trials Partnership (EDCTP), created in 2003, is a partnership of 14 participating European Union (EU) Member States plus Norway and Switzerland, with sub-Saharan African countries. EDCTP aims to accelerate the development of new or improved drugs, vaccines, microbicides and diagnostics against poverty-related diseases (PRDs) including HIV/AIDS, tuberculosis (TB), malaria and neglected infectious diseases (NIDs) [1].

Like other organisations that support research to generate new knowledge for translation into policy and practice, there is a need for EDCTP to assess the output and potential impact of the research that it funds. Although there are many indicators that can be used to measure research progress and knowledge, biomedical publications and their citations are widely used.

Bibliometric methods have been used to analyse publication output and impact for specific disease areas, to quantify the volume of research output and compare the contributions from different institutions, countries and regions [2]. These methods can also be used to map research collaboration at the national, regional and international level and to compare its potential impact. The methods are based on mathematical and statistical techniques and therefore can provide a quantitative assessment of research performance [2].

Journal papers report research work. These papers refer to or ‘cite’ earlier relevant work and the new papers will be cited later, in their turn. The more citations a paper accumulates the more it is considered having ‘impact’. Therefore, citation counts are recognised as a measure of impact, and can be seen as an indicator of the strength of the innovative research from a group, an institution, a country or a region. Most impact indicators do not use simple counts of citations; they use average (normalised) citation counts for defined groups of papers, as individual papers may have varying or unusual citation profiles. Citation rates differ across subject areas and over time. For example, citations in biological sciences occur more rapidly and plateau at a higher level than citations in physical sciences or mathematics, and citation rates are generally higher for natural sciences than for social sciences [3]. In addition, older papers have more time to accumulate citations than more recent ones.

The main objective of this article is to assess the geographical and temporal trends in the publication of research papers on HIV/AIDS, TB, malaria and NIDs by European EDCTP member countries and sub-Saharan African countries through a bibliometric analysis. The secondary objectives of this article are to describe collaboration patterns and to assess the citation impact of research funded by EDCTP.

## Methods

### Data source

Publication data were drawn from the Thomson Reuters Web of Science that annually index the contents of over 12,000 journals worldwide and contains the Science Citation Index. We first drew out all relevant research publications on PRDs using search terms (Table 1) anywhere in their title, abstract or keywords for the period 1 January 2003 to 31 December 2012 (“publication date field”) with citation data up to 31 December 2011. Publications from substantive journal articles, reviews and proceedings papers published in peer-reviewed journals were included in the data extraction; hereafter referred to as ‘papers’. We excluded editorials, meeting abstracts and other types of publications. All identified relevant papers from Web of Science in each disease area were included in the research and matched to PubMed using meta-data (i.e., digital object identifier, author, and source title) [4] [5] to identify relevant epidemiological or clinical research papers. Epidemiological papers were identified using the PubMed Medical Subject Headings (MeSH) where the qualifier contained the term ‘epidemiology’[5]. Clinical trials research was identified using PubMed specific publication types (‘clinical trial’(including phase I-phase IV); ‘controlled clinical trial’ or ‘randomized controlled trial’) or MeSH Headings containing either ‘clinical trial’ or ‘controlled trial’[5].

**Table 1:** Summary of disease areas, infective agents and search terms used.

Disease Areas /Alternative name(s)	Infectious agent	Search term
Human Immunodeficiency Virus (HIV), Acquired Immunodeficiency Syndrome (AIDS)		#1 TS^§^ = ((“human immuno$deficiency virus" OR "acquired immuno$deficiency syndrome" OR "hiv") NOT (feline OR simian OR siv*)), #2 TS = ("aids") NOT #1
Malaria	Plasmodium, Anopheles Mosquito, P. Falciparum, P. Vivax, P. Ovale, P. Malariae	TS = (malaria* OR (plasmodium NOT physarum) OR anopheles OR "black water fever")
Tuberculosis (TB) / Koch's Disease	Mycobacterium tuberculosis (MTB)	TS = (tuberculosis OR "tubercle bacillus" OR "tb infection" OR "pulmonary tb" OR "extrapulmonary tb" OR tuberculin)
**Neglected Infectious Diseases** ^1^		
Echinococcosis / Hydatid Disease	Echinococcus Granulosus, Echinococcus Ultilocularis, Echinococcus Vogeli, Echinococcus Oligarthus	TS = (echinococcosis OR "hydatid disease*" OR echinococcus)
Fascioliasis / Faciolosis Distomatosis	Fasciola Hepatica, Fasciola Gigantica	TS = ((fasciolosis OR fascioliasis OR distomatosis OR "fasciola hepatica" OR "fasciola gigantica") NOT cattle) NOT WC = (veterinary sciences OR agriculture dairy animal science))
Human African Trypanosomiasis / Sleeping Sickness	Glossina Trypanosoma	TS = ((trypanosom* NEAR/5 africa*) OR "sleeping sickness" OR (trypanosom* NEAR/5 tsetse) OR (trypanosom* NEAR/5 human))
Leishmaniasis / Kala azar	Sandfly Lutzomyia	TS = ("sand fly" OR sandfly OR sandflies OR "sand flies" OR leishmaniasis OR leishmania OR phlebotomine OR psychodidae OR kala$azar)
Leprosy / Hansen's Disease	Mycobacterium leprae	TS = (leprosy OR (hansen* NEAR/5 disease*) OR "mycobacterium leprae" OR "erythema nodosum leprosum")
Lymphatic Filariasis / Elephantiasis	Wuchereria Bancrofti, Brugia Malayi, Brugia Timori	TS = ((lymphatic NEAR/5 filariasis) OR elephantiasis OR wuchereria OR "brugia malayi")
Onchocerciasis / River Blindness, Robles' Disease	Onchocerca Volvulus	TS = (onchocerciasis OR "river blindness" OR "onchocerca volvulus")
Rabies		TS = (rabies)
Schistosomiasis / Bilharziosis, Snail Fever, Bilharzia	Some Schistosoma Spp.	TS = (schistosomiasis OR bilharzi* OR "schistosoma mansoni" OR "schistosoma haematobium" OR "schistosoma intercalatum" OR "schistosoma japonicum" OR "schistosoma mekongi")
Soil Transmitted Helminthiasis	Ascaris Lumbricoides Trichuris Trichiura Hookworm	TS = (helminth* OR hookworm* OR "ascaris lumbricides" OR "trichuris trichiura" OR geohelminth OR "necator$americanus" OR "ancylostoma$duodenale")
Trachoma / Granular Conjunctivitis, Egyptian Ophthalmia	Chlamydia Trachomatis	TS = (trachoma)
Yaws / Frambesia Tropica	Treponema Pallidum	TS = (yaws OR treponematos*)

^§^ The search for diseases includes the title, abstract and key words and is denoted as TS = (…)

* This symbol is used to denote any number of characters e.g. network* finds networks, networking, networkers as well as network

$ This symbol denotes a single character e.g. utili$ation finds both utilisation and utilization

^1^ These neglected infectious diseases are the 17 diseases prioritized by the World Health Organization. In reference to the foodborne trematodiases we only included the major foodborne trematodiases prevalent in sub-Saharan Africa (ie Fascioliasis) in the analysis.

To assess the citation impact of research funded by EDCTP, the EDCTP publication database, which includes publications arising to EDCTP-funded research and available in-house (n = 244 papers), was matched to the Web of Science. Subsequently, EDCTP- related papers were also identified by using funding acknowledgment data in Web of Science. This iterative process resulted in a final count of 437 EDCTP-related papers matched to the Web of Science and 258 papers were matched to the 2003–2012 main dataset. Of these, 237 papers were used in the citation analysis.

To conduct region specific analysis, the European EDCTP member countries and sub-Saharan African partner countries were grouped into the respective regions as detailed in Table 2. The data are presented as five-year moving averages (i.e., 2003–2007, 2004–2008, 2005–2009, 2006–2010, and 2007–2011) to smooth out the yearly fluctuations in the number of papers and their citations, allowing for accurate trend analysis.

**Table 2:** EDCTP European member countries and sub-Saharan African partner countries, 2003–2012.

**European EDCTP Member Countries** ^**1**^		**West Africa**	
Austria	AUT	Benin	BEN
Belgium	BEL	Burkina Faso	BFA
Denmark	DNK	Cape Verde	CPV
France	FRA	Côte d'Ivoire	CIV
Finland*	FIN	Ghana	GHA
Germany	DEU	Guinea	GIN
Greece	GRC	Guinea Bissau	GNB
Ireland	IRL	Liberia	LBR
Italy	ITA	Mali	MLI
Luxembourg	LUX	Mauritania	MRT
Netherlands	NLD	Niger	NER
Portugal	PRT	Nigeria	NGA
Spain	ESP	Senegal	SEN
Sweden	SWE	Sierra Leone	SLE
UK	UK	The Gambia	GMB
Norway	NOR	Togo	TGO
Switzerland	CHE		
**Central Africa**		**Southern Africa**	
Angola	AGO	Botswana	BWA
Cameroon	CMR	Comoros	COM
Central African Republic	CAF	Lesotho	LSO
Chad	TCD	Madagascar	MDG
Congo	COG	Malawi	MWI
Democratic Republic of Congo	COD	Mauritius	MUS
Equatorial Guinea	GNQ	Mozambique	MOZ
Gabon	GAB	Namibia	NAM
São Tomé and Príncipe	STP	Seychelles	SYC
**East Africa**		South Africa	ZAF
Burundi	BDI	Swaziland	SWZ
Eritrea	ERI	Zambia	ZMB
Ethiopia	ETH	Zimbabwe	ZWE
Kenya	KEN		
Rwanda	RWA		
Somalia	SOM		
South Sudan	SSD		
Sudan	SDN		
Tanzania	TZA		
Uganda	UGA		

^1^ European EDCTP Member Countries: Members of the EDCTP-EEIG and represented in the EDCTP General Assembly.

* Finland had observer status in the EDCTP-EEIG

### Assignment of papers

A paper was assigned to each country and each organisation that appeared at least once for any author on the paper. A paper was counted only once for each country and organisation, irrespective of the number of variations in the addresses. For example, a paper with four authors, all from the same country, with two authors from the same organisation and the other two from two different organisations would be assigned to the country only once and once to each of the three different organisations to avoid double-counting of either institutional or country-level data. Of note, international, governmental and non-governmental organisations were assigned according to the country given by the author. This includes organisations such as the World Health Organization (WHO) (headquartered in Geneva, Switzerland). The source of funding has only been indexed in Web of Science consistently since mid-2008, although it is generally acknowledged in publications. For this reason, and because authors do not always present their organisation in the same way, algorithms were used to unify the variants used for the various funding sources and organisations. All papers that acknowledged EDCTP were attributed to EDCTP; as the authors may have chosen to acknowledge EDCTP without having received funding support from EDCTP, these papers were compared with EDCTP’s records to confirm if funding had been received from EDCTP. Papers identified in the initial extraction were matched with publication data from EDCTP to produce a subset of papers for which EDCTP funding could be confirmed. Only papers for which EDCTP financial support was confirmed were included in analysis of the impact of EDCTP-funded research.

### Standard bibliometric indicators

Five bibliometric indicators were considered in our analysis; the first four indicators are based at paper level and the fifth at journal level.

#### Citation count

This is defined as the number of times that a citation has been recorded for a given publication since it was published. While not all citations are necessarily recorded or abstracted in the Web of Science, the material abstracted by Thomson Reuters is estimated to attract about 95% of global citations. The citation count declines in the most recent years of any time-period as papers would have had less time to accumulate citations.

#### Citation impact

Also called ‘citations per paper’ is an index of academic or research impact (as compared with economic or social impact) and is calculated by dividing the sum of citations by the total number of papers in any given dataset. Thus, for a single paper, the citation impact is the same as its citation count. The citation impact can be calculated for papers within a specific research field, such as HIV/AIDS, or for a specific institution, group of institutions, or a specific country.

#### Normalised citation impact (NCI)

This indicator corresponds to the relative number of citations to publications from a specific unit (i.e. institution, country, research field), compared to the world average of citations to publications of the same document type, age and subject area. The most common normalisation factors are the average citations per paper for the year and either the field or journal category in which the paper was published. This normalisation is also referred to as ‘rebasing’ the citation count. An NCI value of one represents performance at par with world average, values above one are considered above average and values below one are considered below average [4]. As an example, 0.9 means that a unit’s publications are cited 10% below average and 1.2 that they are cited 20% above average. The NCI is a valuable indicator in bibliometrics as it allows comparisons between entities of different sizes and different subject mixes.

#### Impact profiles

While the NCI is a useful indicator of research performance, it does not describe the underlying distribution of citation impact. Adams et al [6] developed an impact profile methodology to address this limitation. An Impact Profile visualises a citation impact distribution as it shows the proportion of papers that are uncited and the proportion that lie in each of eight categories (i.e. > 0<0.125; ≥0.125<0.25; ≥0.25<0.5; ≥0.5<1; ≥1<2;≥2<4; ≥4<8, and ≥ 8) of relative citation rates, normalised (rebased) to world average (1.0) [6]. An Impact Profile allows the strengths and weaknesses of published output to be assessed in comparison with the world average and a reference profile. This provides much more information about the basis and structure of research performance at institutional or country level than conventionally reported averages in citation indices. Impact Profiles for EDCTP-funded papers in each disease area were calculated as described by Adam et al [6] and compared with papers from collaborative research between Europe and sub-Saharan Africa (i.e., the reference profile) and the world average. For the purpose of this study, papers that were cited ≥4 times the relevant world average were defined as highly-cited. To note, “output with high-end impact (i.e., the percentage of outputs that excess eight times the world average impact) is most likely to contribute to research and economic innovation and competitiveness” [6].

#### Journal impact factor (JIF)

Similar to the normalised citation impact, the average number of citations per paper can be used to indicate the impact/importance of a journal. The JIF used in the analysis are from the Thomson Reuters’ Journal Citation Report 2011 [7]. The JIF is a measure of the frequency with which the ‘average article’ in a journal has been cited in a particular year or period [8]. The annual JIF is a ratio between the number of citations and the number of citable items published in a recent period. Thus, the JIF is calculated by dividing the number of current year citations by the number of articles published in the specific journal during the previous two years [8]. It is important to note that citation rates vary between research fields and publication types, and this affects the JIF [3]. Ranking the journals within subject categories can adjust for differences in citation rates between research fields. For example, the 2011 impact factor for the journal *Vaccine* was 3.766; it was ranked 41 out of 138 journals in the immunology’ Web of Science journal category, which placed it in the second quartile. It was also ranked 24 out of 110 journals in the ‘research and experimental medicine’ Web of Science journal category and therefore was in the first quartile for this category. In the analysis, the highest quartile of a journal was taken into consideration when it appeared in more than one journal category (i.e. the first quartile in the example above).

### Collaboration maps

Co‐authorship is an index of research collaboration. The share of output that was collaborative across the disease areas were analysed using the address data associated with the publication. The software Wolfram Mathematica was used to create the maps and to produce a visual representation of the citation impact between EDCTP member countries (institutions) and sub-Saharan Africa and within sub-Saharan Africa. Disease burden data were obtained from the World Health Organisation (WHO) Global Burden of Disease estimates [9]. The overall burden of disease was assessed using the disability-adjusted life year (DALY). DALY rates are expressed per 100,000 population using 2004 population estimates. The updated WHO global health estimates were only published after the completion of this study and were therefore not used.

## Results

We identified 290,539 publications produced between 2003 and 2012 in the field of PRDs of which 202,494 papers (69.7%) were used in the citation analyses (2003–2011) (S1 Diagram). We analysed 94,827 (46.8%) papers on HIV/AIDS, 33,621 (16.6%) on TB, 29,714 (14.7%) on malaria and 44,332 (21.9%) on NIDs. Although papers can cover more than one disease, there was little overlap between the disease areas in our dataset. Only 71 papers in our PRD dataset related to two or more other diseases. The main overlap was between HIV/AIDS and TB – 14.8% of TB research papers were also in the HIV/AIDS dataset and 5.3% HIV/AIDS research papers were also related to TB research, and 12.2% of malaria research papers were also in the NIDs dataset, while 8.2% of the research in NIDs also related to research in malaria.

The match rate between the Web of Science and PubMed to identify relevant papers in epidemiological or clinical research papers was 87.3% (n = 82,798 papers) for HIV/AIDS; 88.3% (n = 29,700 papers) for TB; 86.3% (n = 25,644 papers), and 85.2% (n = 37,758 papers) for NIDs. Epidemiological and clinical trials research papers were the focus of a much higher percentage of research outputs from sub-Saharan Africa (N = 10,791 papers; 53.1%), than globally (46,402 papers; 26.4%).

For all PRDs covered, there was a clear trend of increased publication in open-access journals. By 2007–2011 the number of HIV/AIDS papers published in open access journals increased from 7.2% of papers in 2003–07 to 14.7%. For the TB papers the increase was from 10.2% to 19.4%; for the malaria papers it was from 12.2% to 26.6%, and for the NIDs papers it was from 13.3% to 23.4%.

To calculate the Impact Profiles we identified in the Web of Science database 237 papers that reported results from EDCTP-funded research (S1 Diagram). Overall, 221 (93.2%) of these papers were published in journals ranked in the top two JIF quartiles, with 166 paper (70%) published in journals in the top quartile. Almost 80% of the malaria papers (46/58 papers) were published in journals in the top quartile compared with 66.7% of the papers on HIV/AIDS (96/144 papers), 66.7% of the papers on TB (66/99 papers) and 66.1% of the papers on HIV/TB co-infections (41/62 papers).

### HIV/AIDS research

**Fig 1:**
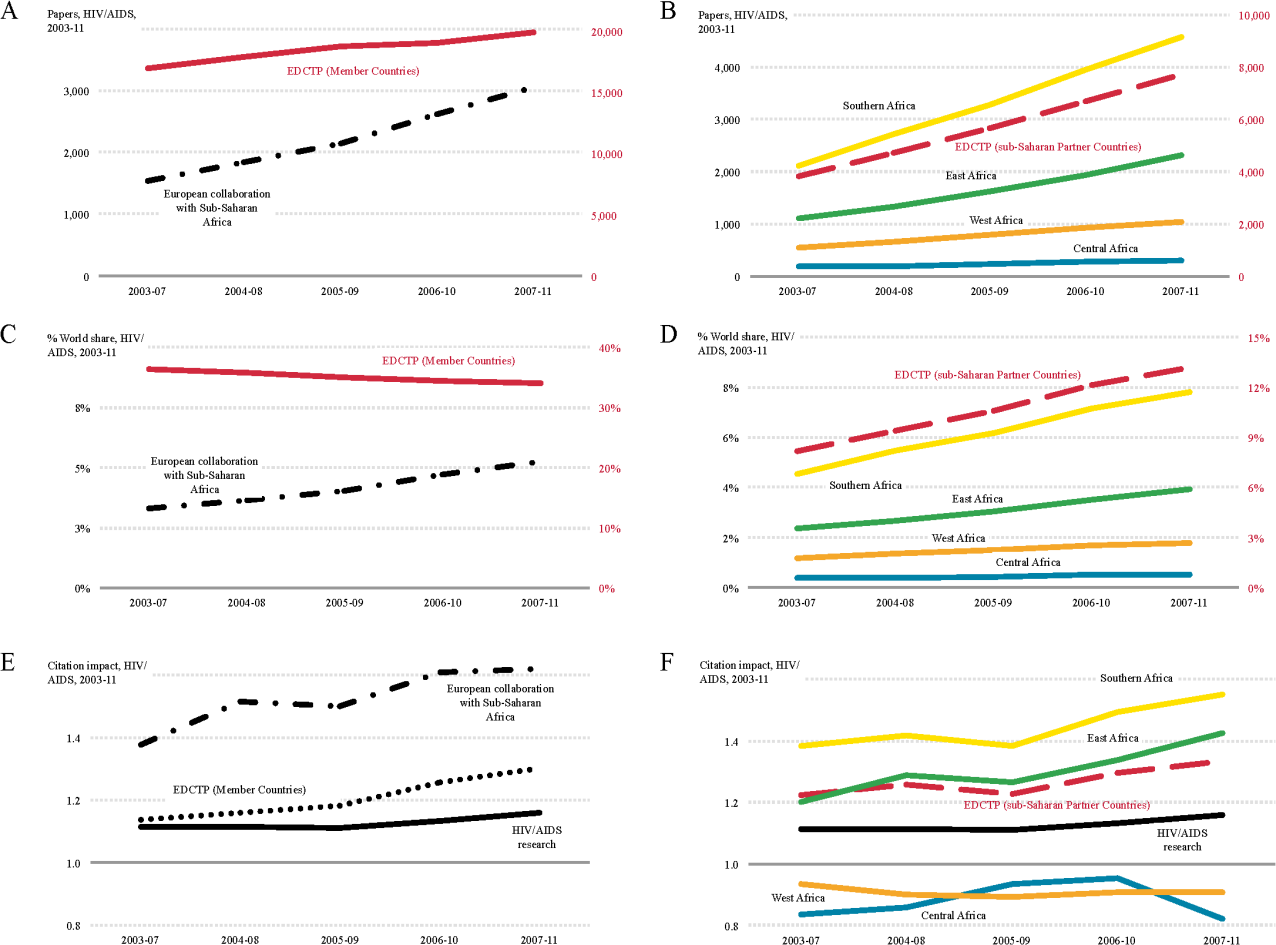
Trends in HIV/AIDS research output for 2003–2011. Research output (number of publications) in HIV/AIDS for EDCTP member countries (right-hand red y-axis) and European collaboration with sub-Saharan Africa (left-hand black y-axis) (A) and for EDCTP Sub-Saharan Partner Countries (right-hand red y-axis) and the four regions in sub-Saharan Africa (left-hand black y-axis) (B). World share in HIV/AIDS publications for EDCTP member countries (right-hand red y-axis) and European collaboration with sub-Saharan Africa (left-hand black y-axis) (C) and for EDCTP Sub-Saharan Partner Countries (right-hand red y-axis) and the four regions in sub-Saharan Africa (left-hand black y-axis) (D). Citation impact for HIV/AIDS publications from EDCTP member countries d European collaboration with sub-Saharan Africa (E) and for EDCTP Sub-Saharan Partner Countries and the four regions in sub-Saharan Africa (F); the world average normalised citation impact for HIV/AIDS is shown as a solid black line in (E) and (F) for comparison.

Although the output for HIV/AIDS increased over the study period in Europe (17.2%) and sub-Saharan Africa (102.4%), the percentage of the overall world output decreased for Europe (from 36.4% in 2003–2007 to 34.0% in 2007–2011), while the percentage of the overall world output for sub-Saharan African countries increased (from 8.2% in 2003–2007 to 13.2% in 2007–2011), particularly for Southern Africa (from 4.5% in 2003–2007 to 7.8% in 2007–2011) (Fig 1).

The output from European collaboration with sub-Saharan African countries nearly doubled over the study period and the share of this collaborative research as percentage of the overall world production increased from 3.3% in 2003–2007 to 5.2% in 2007–2011 (Fig 1). The normalised citation impact (NCI) of this collaborative research was higher (NCI: 1.62) than for European (NCI: 1.30) or sub-Saharan African countries (NCI: 1.33) separately.

**Fig 2:**
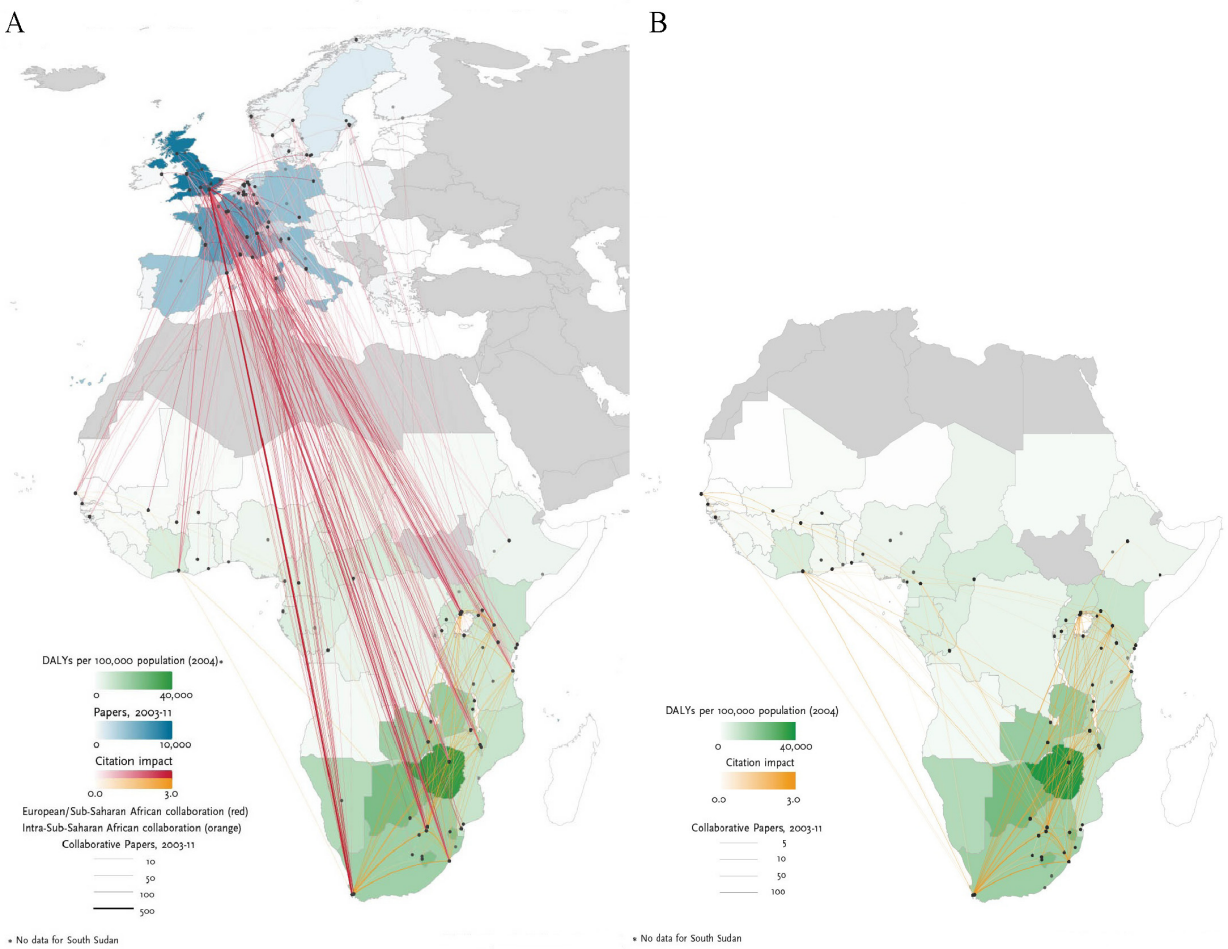
HIV/AIDS burden of disease and collaboration research patterns. Left panel A: HIV disease burden in Sub-Saharan Africa, research output in Europe and their collaborative research links and citation impact between Europe and Sub-Saharan Africa. Right-hand panel B: HIV disease burden and collaborative research links and citation impact within Sub-Saharan Africa. Graphs were produced by Thomson Reuters (*Evidence*).

Overall, institutions in the United Kingdom (UK) (2080 papers) and France (706 papers) were the leading European collaborating partners with sub-Saharan Africa in HIV/AIDS research and this research was well-cited (NCI: 1.83 and 1.64, respectively) followed by collaborative research with institutions in the Netherlands (448 papers, NCI: 1.48) and Belgium (389 papers, NCI: 1.51) (Fig 2). Output, collaboration and normalised citation impact in HIV/AIDS research within sub-Saharan Africa was led by Southern (NCI: 1.55) and East Africa (NCI: 1.42) whereas research output, collaboration and normalised citation impact was lower in and between West (NCI: 0.91) and Central Africa (NCI: 0.82) (Fig 2). These results partly reflect the disease burden as countries with higher DALYs (disability-adjusted life year) were more active in this research area although some regional variation was observed (Fig 2).

#### Clinical trials and epidemiology research

Of the 8,955 sub-Saharan HVV/AIDS research papers from which PubMed meta data were available, 3,934 papers (43.9%) were identified as epidemiology research and 1.080 (12.1%) as clinical trials. These papers were mostly published by researchers in Southern (55.3%) and East Africa (34.0%).

#### Impact profiles

EDCTP-funded research papers in HIV/AIDS (n = 144) had higher normalised citation impact (NCI: 3.24) compared with benchmarks for European collaboration with sub-Saharan Africa (NCI: 1.55) or the world average impact (NCI: 1.14). Furthermore, among the 144 HIV/AIDS papers, 18.7% (n = 27) were highly cited (NCI categories: ≥4-<8 and ≥8); these were 9 papers related to HIV/AIDS and 18 papers related to HIV/TB co-infections. This proportion of highly cited papers was greater compared to European-sub-Saharan Africa (9.6%) or world benchmarks (5.8%) (S1 Fig).

### TB research

During the study period sub-Saharan African TB research output increased by 81% and the region’s share of global TB research increased from 8.0% in 2003–2007 to 10.3% by 2007–2011. The normalised citation impact of this research was well above the world average rising from 1.18 in 2003–2007 to 1.67 in 2007–2011. Only the normalised citation impact of papers from Central Africa (NCI: 0.86, 2007–2011) was below the world average. This increase in sub-Saharan African output and impact was mainly driven by Southern Africa, which accounted for nearly 64% of the entire sub-Saharan African TB research output and which was highly cited (NCI: 1.97,2007–2011).

**Fig 3:**
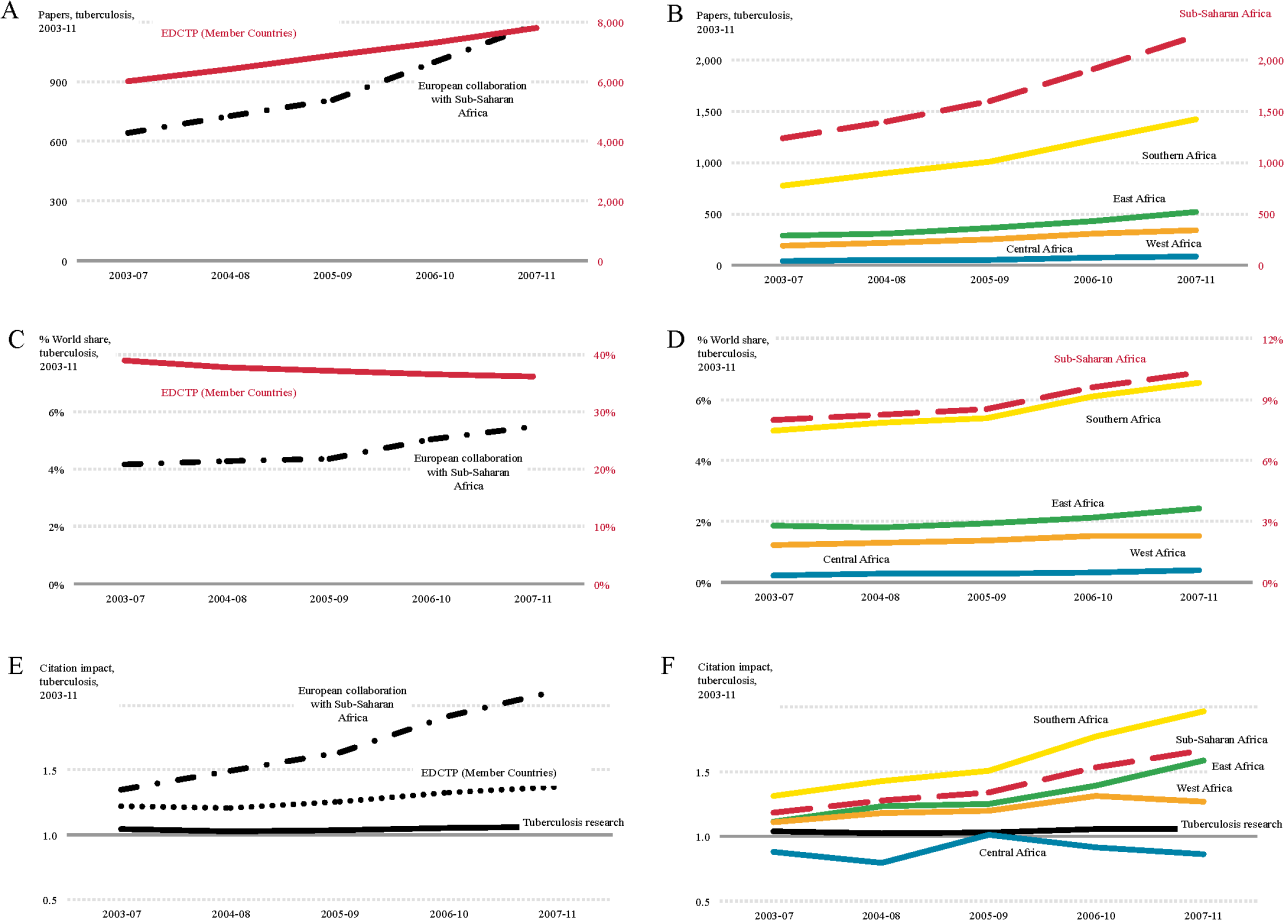
Trends in TB research output for 2003–2011. Research output (number of publications) in TB for EDCTP member countries (right-hand red y-axis) and European collaboration with sub-Saharan Africa (left-hand black y-axis) (A) and for EDCTP Sub-Saharan Partner Countries (right-hand red y-axis) and the four regions in sub-Saharan Africa (left-hand black y-axis) (B). World share in TB publications for EDCTP member countries (right-hand red y-axis) and European collaboration with sub-Saharan Africa (left-hand black y-axis) (C) and for EDCTP Sub-Saharan Partner Countries (right-hand red y-axis) and the four regions in sub-Saharan Africa (left-hand black y-axis) (D). Citation impact for TB publications for EDCTP member countries and European collaboration with sub-Saharan Africa (E) and for EDCTP Sub-Saharan Partner Countries and the four regions in sub-Saharan Africa (F); the world average normalised citation impact for TB is shown as a solid black line in (E) and (F) for comparison.

It would seem that a shift in focus towards HIV/TB co-infection was responsible for the trend of more research output from sub-Saharan Africa, particularly in more recent years (Fig 3).

In Europe, the TB research output increased by 29.7% but the percentage share of world output decreased by 7.4% during the study period. Nevertheless, Europe produced over a third of the global research output in TB and this research was highly cited (NCI: 1.37, 2007–2011).

**Fig 4:**
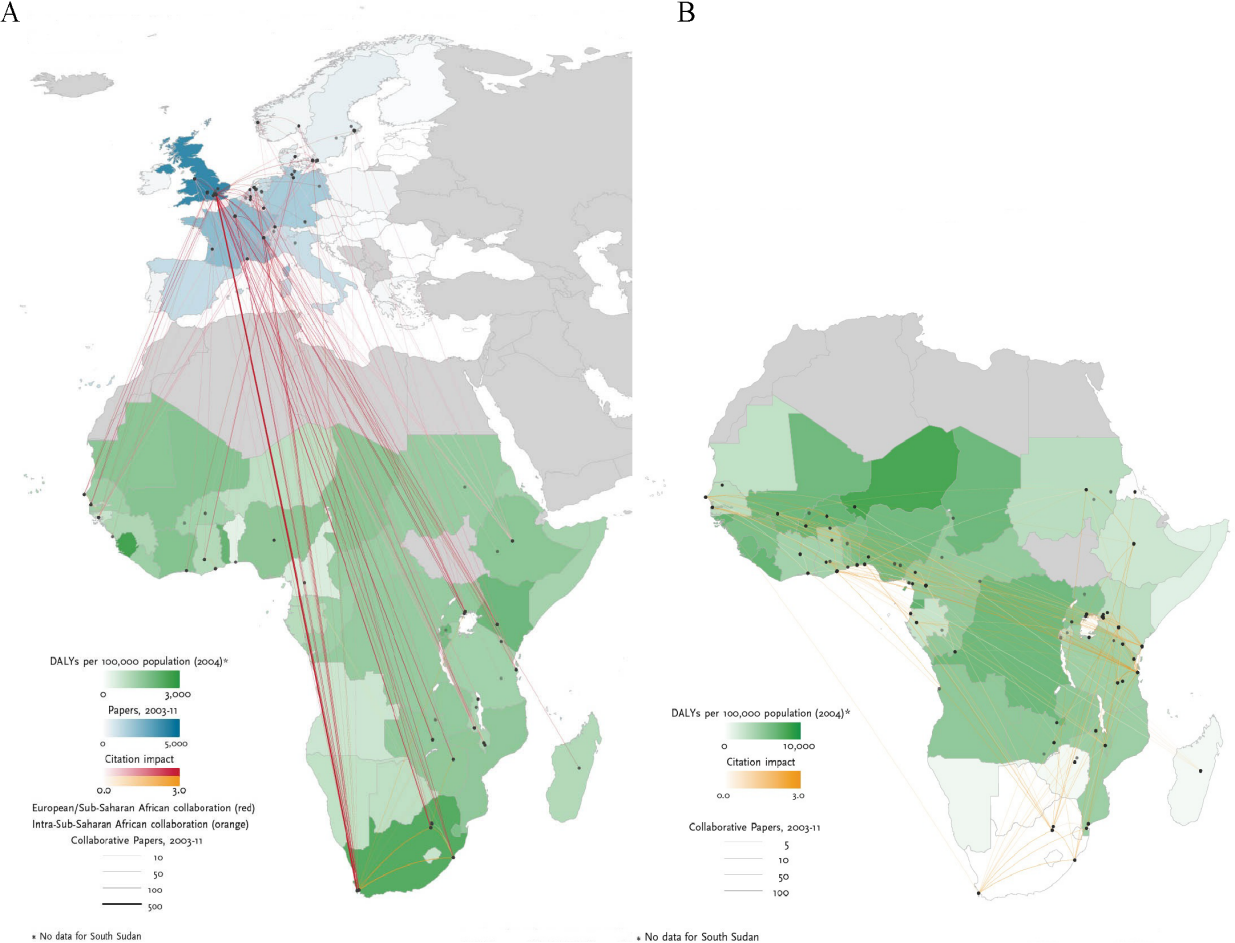
TB burden of disease and collaboration research patterns. Left-hand panel A: TB disease burden in Sub-Saharan Africa, research output in Europe and their collaborative research links and citation impact between Europe and Sub-Saharan Africa. Right-hand panel B: TB disease burden and collaborative research links and citation impact within Sub-Saharan Africa. Graphs were produced by Thomson Reuters (*Evidence*).

The collaboration between Europe and sub-Saharan Africa increased by 84.6% between 2003 and 2011, driven by multidisciplinary HIV/AIDS and TB research which was well-cited (NCI: 2.11). Similar to HIV/AIDS research, leading European collaborating partners with sub-Saharan Africa (mainly Southern and East Africa) in TB research were institutions in the UK (809 papers, NCI: 2.19), France (267 papers, NCI: 1.80), the Netherlands (222 papers, NCI: 1.96), and Switzerland (224 papers, NCI: 3.63) (Fig 4). As shown in Fig 4, TB research efforts are not correlated with burden of disease, except for South Africa, and the research collaboration on TB within sub-Saharan Africa is weak.

#### Clinical trials and epidemiology research

Of the 2,833 sub-Saharan TB research papers from which PubMed meta data were available, 1,206 papers (42.6%) were identified as epidemiology research and 219 (7.8%) as clinical trials. These papers were also mostly published by researchers in Southern (59.2%) and East Africa (27.4%).

#### Impact profiles

EDCTP-funded TB papers had a normalised citation impact that was more than four-times the world average (NCI: 4.08). This was much higher than the normalised citation impact of other European collaborations with sub-Saharan Africa (NCI: 1.88) or world average impact (NCI: 1.05). The curve representing the EDCTP TB Impact Profile (S2 Fig) was located well to the right of the others, indicating that the normalised citation impact of EDCTP TB research was exceptionally high with 26.2% (n = 26) of EDCTP-funded papers being highly cited (NCI categories: ≥4-<8 and ≥8), including 8 TB papers and 18 papers related to HIV/TB co-infections. This proportion of highly cited papers was greater compared to the benchmark for European collaboration with sub-Saharan Africa (11.8%) or the world average (5.2%). Of note, EDCTP-funded research papers on HIV/TB co-infections were cited more than five-times more often than the world average (NCI: 5.1), significantly higher than benchmarks for European collaboration with sub-Saharan Africa (NCI: 2.22) and global research (NCI: 1.35).

### Malaria research

**Fig 5:**
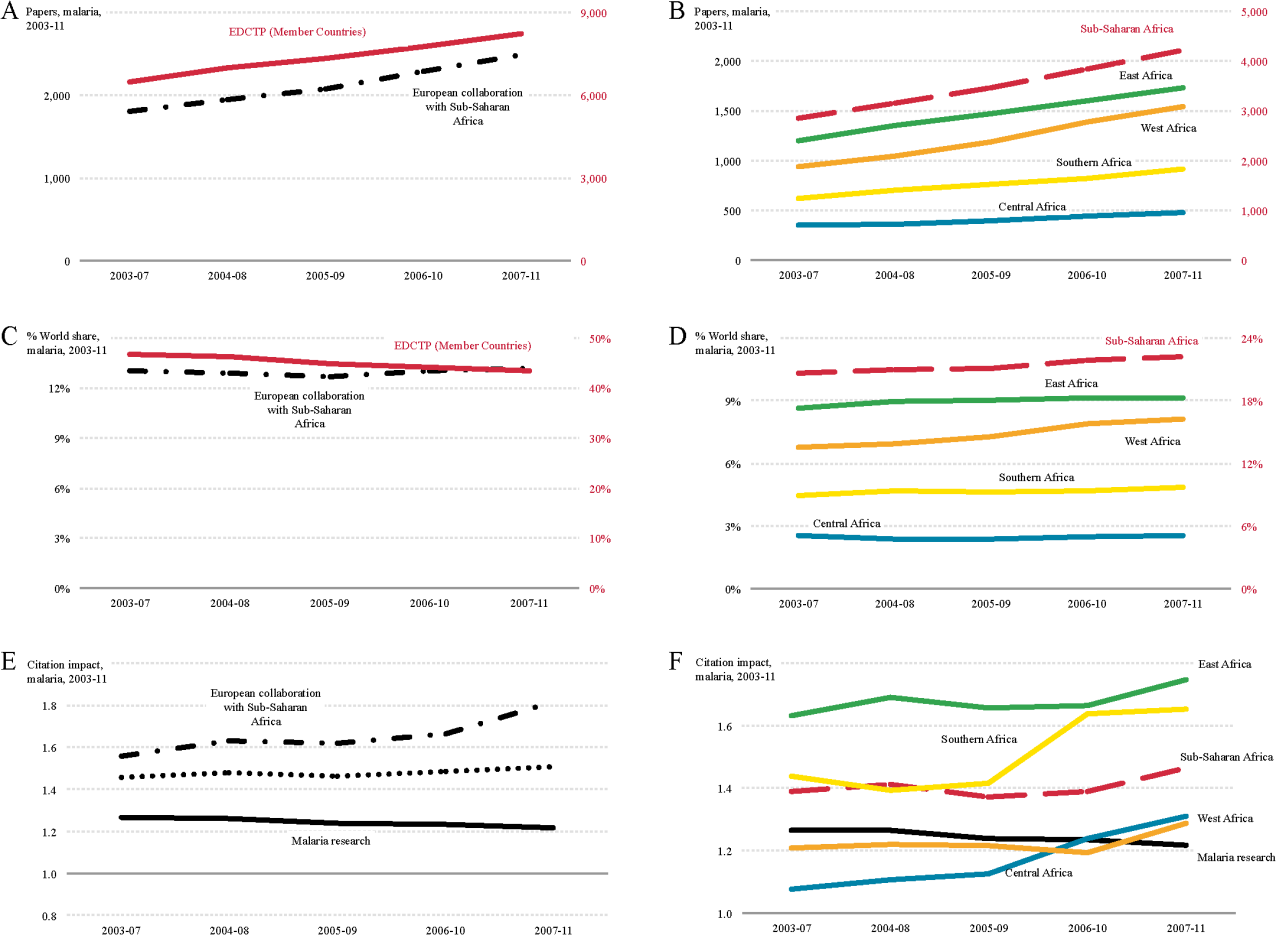
Trends in malaria research output for 2003–2011. Research output (number of publications) in malaria for EDCTP member countries (right-hand red y-axis) and European collaboration with sub-Saharan Africa (left-hand black y-axis) (A) and for EDCTP Sub-Saharan Partner Countries (right-hand red y-axis) and the four regions in sub-Saharan Africa (left-hand black y-axis) (B). World share in malaria publications for EDCTP member countries (right-hand red y-axis) and European collaboration with sub-Saharan Africa (left-hand black y-axis) (C) and for EDCTP Sub-Saharan Partner Countries (right-hand red y-axis) and the four regions in sub-Saharan Africa (left-hand black y-axis) (D). Citation impact for malaria publications for EDCTP member countries and European collaboration with sub-Saharan Africa (E) and for EDCTP Sub-Saharan Partner Countries and the four regions in sub-Saharan Africa (F); the world average normalised citation impact for malaria is shown as a solid black line in (E) and (F) for comparison.

Over the study period, the European share of the world output for malaria research decreased slightly (from 46.7% to 43.4%), while it remained stable in sub-Saharan Africa (~21%), despite increased numbers of papers in both regions (27.2% in Europe; 47.6% in sub-Saharan Africa) (Fig 5). East Africa (9.1%) and West Africa (8.1%) lead in sub-Saharan Africa in terms of share of global malaria research output; the normalised citation impact for East Africa (NCI: 1.75) was the highest but Southern Africa (NCI: 1.65) was almost as high (Fig 5).

**Fig 6:**
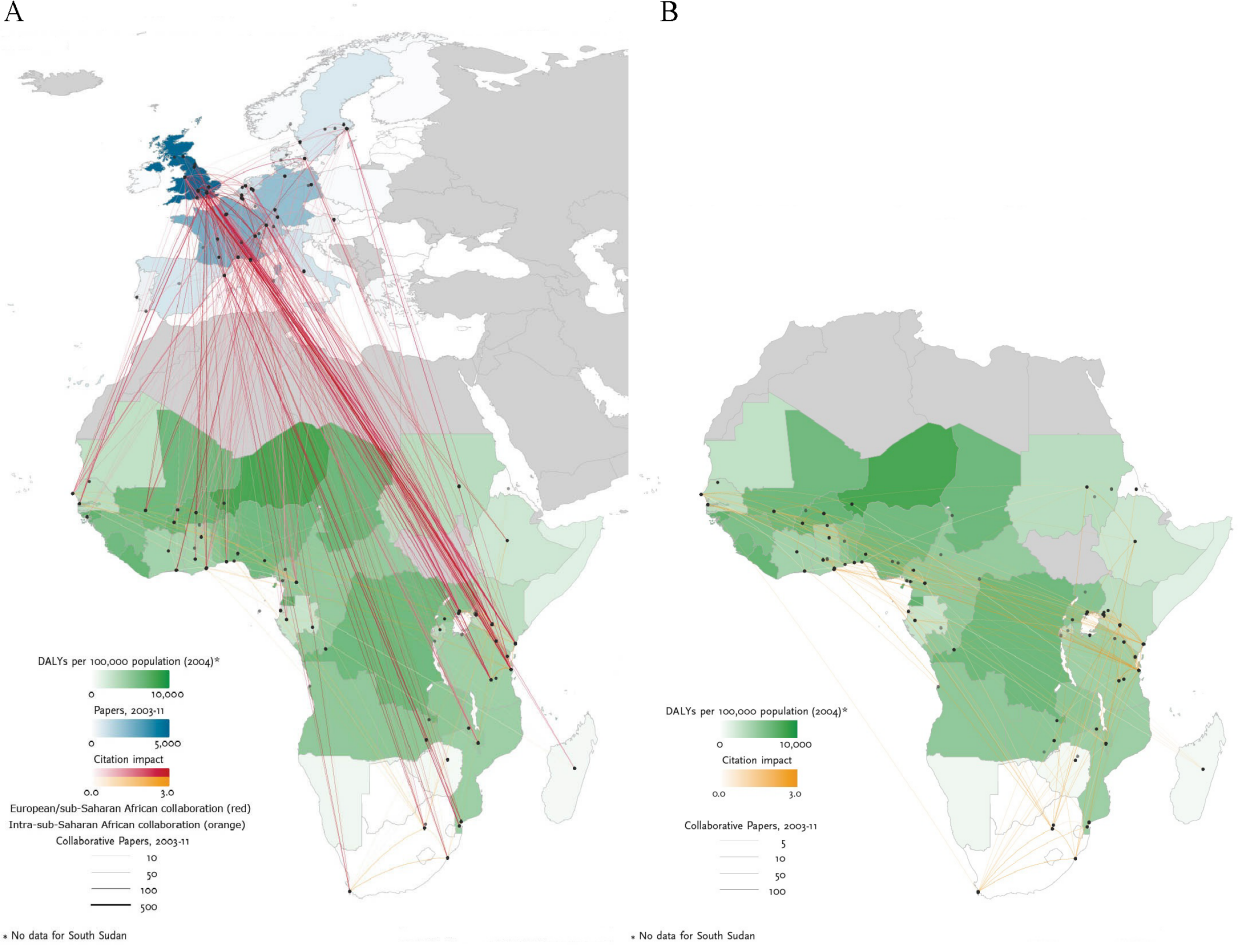
Malaria burden of disease and collaboration research patterns. Left-hand panel A: malaria disease burden in Sub-Saharan Africa, research output in Europe and their collaborative research links and citation impact between Europe and Sub-Saharan Africa. Right-hand panel B: malaria disease burden and collaborative research links and citation within Sub-Saharan Africa. Graphs were produced by Thomson Reuters (*Evidence*).

At the end of the study period the normalised citation impact for collaborative African-European research was 1.81, which was higher than the respective figures for either European (NCI: 1.51) or sub-Saharan African research (NCI: 1.47) (Fig 5). In malaria research, the leading European collaborating partners with sub-Saharan Africa were institutions in the UK (1,836 papers, NCI: 2.19), France (739 papers, NCI: 1.37), Switzerland (552 papers, NCI: 2.29), and Germany (494 papers, NCI: 1.24) (Fig 6). As shown in Fig 6, there is little correlation between burden of disease and malaria research output in sub-Saharan Africa.

#### Clinical trials and epidemiology research

Of the 5,672 sub-Saharan malaria research papers from which PubMed meta data were available, 2,047 papers (36.1%) were identified as epidemiology research and 762 (13.4%) as clinical trials. This research was mostly published by researchers in East (48.1%) and West Africa (34.9.4%).

#### Impact profiles

The normalised citation impact of EDCTP-funded malaria papers was 1.70. Whilst this was higher than the benchmark for global research in malaria (NCI: 1.24), it was comparable to the benchmark for European collaboration with sub-Saharan Africa (NCI: 1.72). Among the 58 malaria papers, 8.6% (n = 5) were highly cited (NCI categories: ≥4-<8 and ≥8). This proportion of highly cited papers was slightly lower compared to the European-sub-Saharan Africa benchmark (9.4%) but higher than the benchmark for global research (6.3%) (S3 Fig).

### NIDs research

**Fig 7:**
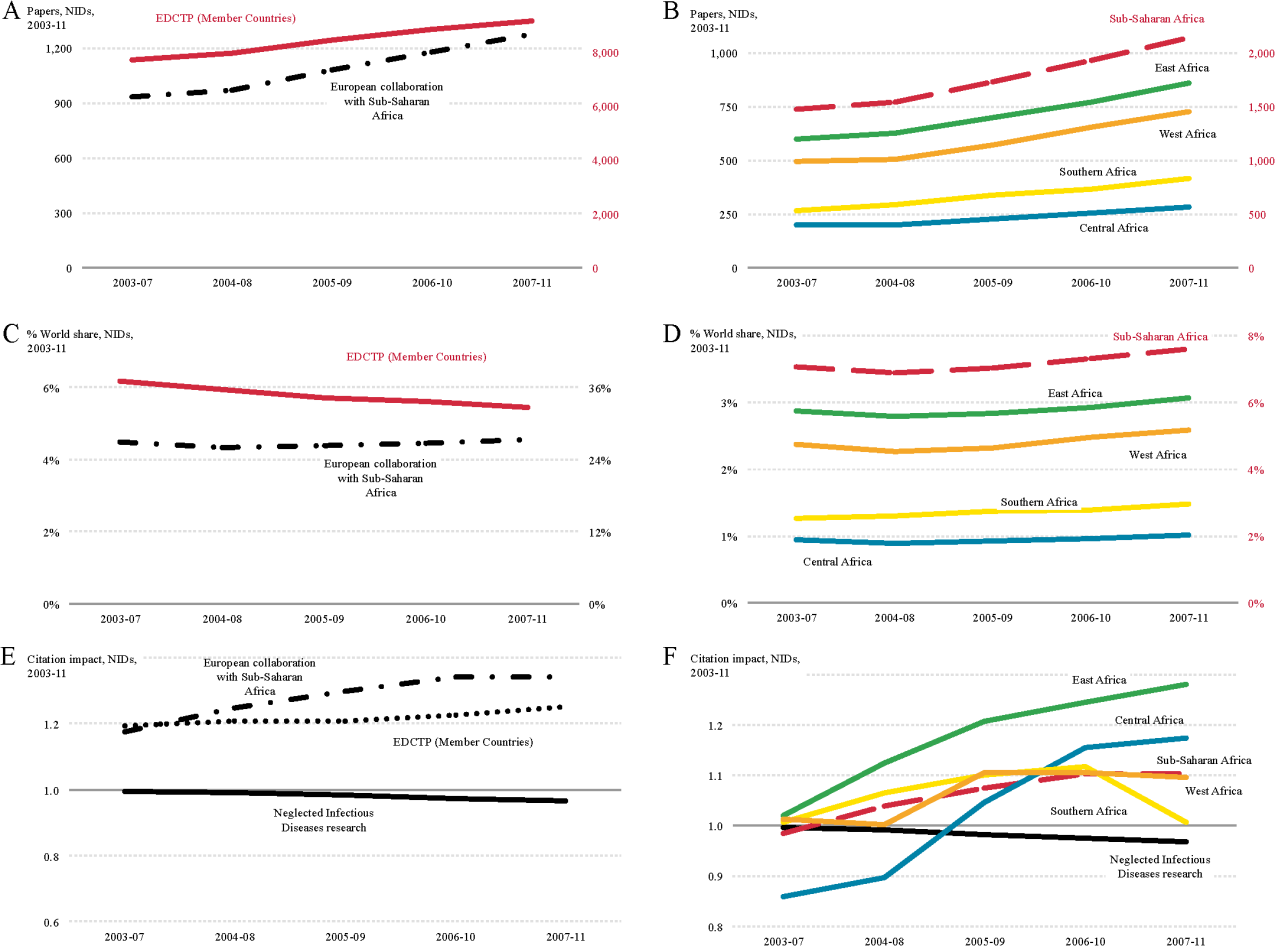
Trends in neglected infectious disease (NID) research output for 2003–2011. Research output (number of publications) in NIDs for EDCTP member countries (right-hand red y-axis) and European collaboration with sub-Saharan Africa (left-hand black y-axis) (A) and for EDCTP Sub-Saharan Partner Countries (right-hand red y-axis) and the four regions in sub-Saharan Africa (left-hand black y-axis) (B). World share in NID publications for EDCTP member countries (right-hand red y-axis) and European collaboration with sub-Saharan Africa (left-hand black y-axis) (C) and for EDCTP Sub-Saharan Partner Countries (right-hand red y-axis) and the four regions in sub-Saharan Africa (left-hand black y-axis) (D). Citation impact for NID publications for EDCTP member countries and European collaboration with sub-Saharan Africa (E) and for EDCTP Sub-Saharan Partner Countries and the four regions in sub-Saharan Africa (F); the world average normalised citation impact for NID is shown as a solid black line in (E) and (F) for comparison.

**Fig 8:**
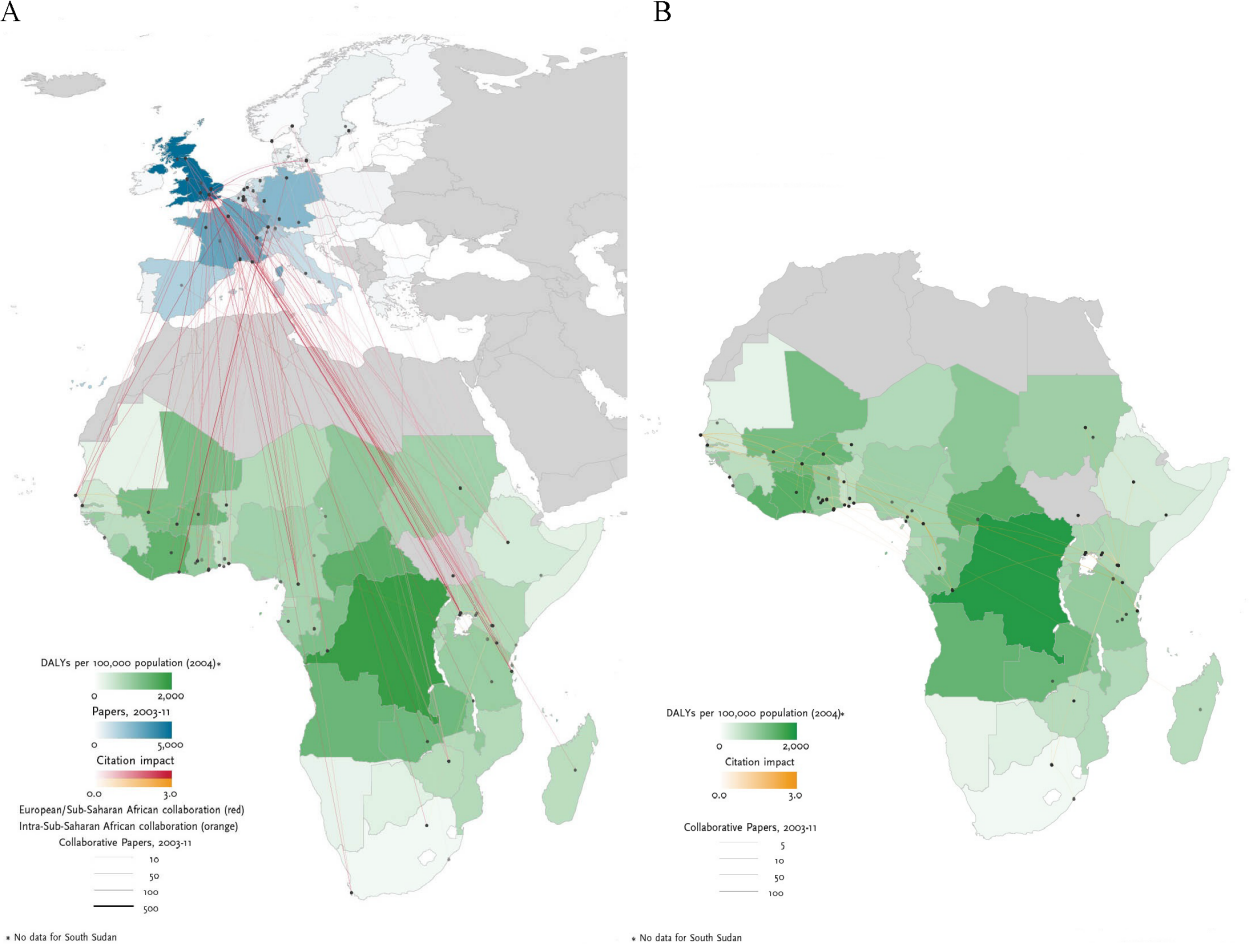
Neglected infectious disease (NID) burden of disease and collaboration research patterns. Left-hand panel A: NID disease burden in Sub-Saharan Africa, research output in Europe and their collaborative research links and citation impact between Europe and Sub-Saharan Africa. Right-hand panel B: NID disease burden and collaborative research links and citation impact within Sub-Saharan Africa. Graphs were produced by Thomson Reuters (*Evidence*).

Compared with HIV, TB and malaria the research output on NIDs was lower during the study period and the research was less well cited (Fig 7). Again, despite increased numbers of papers in both regions (Europe 18.8%; sub-Saharan Africa 45.4%), the share of the world output for NID research decreased in Europe (37.1% to 32.6%) while it remained stable, at about 7%, in sub-Saharan Africa over the study period (Fig 7). Although the citation rates of NID research in sub-Saharan Africa were modest (NCI: 1.10 vs. 1.25 for European countries), the normalised citation impact of research from Central Africa in particular increased, but also from West and East Africa, suggesting more recent research activity in these regions. The normalised citation impact for European collaboration with sub-Saharan Africa was 1.34 at the end of the study period (Fig 7). Leading European collaborating partners with sub-Saharan Africa in NID research were institutions in the UK (882 papers, NCI: 1.53), France (411 papers, NCI: 1.20), Switzerland (273 papers, NCI: 1.98), Germany (245 papers, NCI: 1.07) and Belgium (238 papers, NCI: 1.61). Some collaborative research between institutions in West, Central and East Africa exists but these links were not very strong (Fig 8).

#### Clinical trials and epidemiology research

Of the 2,885 sub-Saharan research papers on NIDs from which PubMed meta data were available, 1,332 papers (46.2%) were identified as epidemiology research and as 216 (7.5%) clinical trials. Similar to malaria, these papers were also mostly published by researchers in East (48.1%) and West Africa (34.9.4%).

## Discussion

Our results show that since 2003 the total research output in PRDs and their associated normalised citation impact and world share from sub-Saharan Africa has risen substantively, particularly in HIV/AIDS and TB, and principally involving Southern and East Africa. The research output in PRDs from sub-Saharan Africa mainly includes clinical and epidemiological research. Furthermore, although European research output generally has increased, its share of the global output for PRDs has dropped. However, the overall output from European research collaboration with sub-Saharan Africa for PRDs has increased over the period studied and the normalised citation impact of this collaborative research is generally higher than that for either European or sub-Saharan African research not involving North-South collaboration. The research output from EDCTP-funded research projects has a higher normalised citation impact for both HIV/AIDS and TB and more than 90% of the papers from EDCTP-funded projects are published in high impact journals (i.e. in the first and second quartile of journals by journal impact factor in their Web of Science journal category).

Although sub-Saharan Africa assumes the heaviest burden of PRDs its share of global PRD research output is still relatively modest compared with European EDCTP member countries that have a much higher research output in this area [10]. For the period 2007–2011, Europe accounted for approximately one-third of the world’s research output on HIV/AIDS, TB and NIDs and 43% on malaria. However, the fall in the European share of the global research output on PRDs is due to the rising research output from BRICK (i.e. Brazil, Russia, India, China and South Korea) economies, in addition to the growth in research output from sub-Saharan Africa [11–13]. For the period 2007–2011, sub-Saharan Africa’s share of global research was higher for malaria (22%) than for the other PRDs (HIV/AIDS 13.2%; TB 10.3%; NIDs 7.6%). Since 2003, the African research output on PRDs has steadily increased, although not uniformly for all diseases or geographic areas. Sub-Saharan Africa’s research output on HIV/AIDS and TB grew faster than that for malaria and NIDs and while Southern Africa dominated the sub-Saharan African HIV/AIDS and TB research output, Eastern Africa dominated the sub-Saharan African malaria and NIDs research output. In contrast, during the study period, Central Africa had a low level of research output for PRDs despite being a region with a high disease burden, particularly for malaria and NIDs [14]. However, although there were a low number of papers, their normalised citation impact for malaria (NCI: 1.31 vs. 1.22) and NIDs (NCI: 1.17 vs. 0.97) is above the world average and thus not insignificant. The observed trend in research output from sub-Saharan Africa suggests that sustained support and funding by governments, development partners, private foundations and public partnerships, such as EDCTP, to address Millennium Development Goal 6 to combat HIV/AIDS, malaria and other diseases is having a positive impact progressively.

A very large share of sub-Saharan African research on PRDs is a result of international collaboration. For 2007–2011, 40%, 53%, 59%, and 60% of clinical research on HIV/AIDS, TB, malaria and NIDs, respectively was conducted in collaboration with European EDCTP member countries. The United Kingdom and France have traditionally been part of these collaborations due to their historic colonial ties with many countries in sub-Saharan Africa. The collaboration trends show that several European countries are increasing their research interests in PRDs with increasing collaboration among themselves and sub-Saharan African countries, particularly Belgium, Denmark, the Netherlands, Sweden and Switzerland. This could be attributed partly to funding instruments like the EDCTP programme that actively promotes cross-national research through North-South, North-North and South-South networking.

As highlighted by the World Bank’s recent publication, a high collaboration rate reflects the “noteworthy effort and interest of academia outside of Africa to support sub-Saharan Africa’s research capacity” [15]. This report pointed out that “international collaboration is highly instrumental in raising the impact of sub-Saharan Africa’s publications” [15]. Our results corroborate this statement as illustrated in the example of Central Africa, where few research groups operate, our results show that their research activities are highly collaborative (HIV/AIDS: ≥40%; TB; ≥53%; malaria and NIDs ≥60%) resulting in normalised citation impact for malaria and NIDs that are above the world average. This trend was also seen for research activities funded by EDCTP. The normalised citation impact of EDCTP-funded collaborative research on PRDs between European EDCTP member countries and sub-Saharan African countries was higher than the respective figures for either European or sub-Saharan African research. These findings suggest that collaborative research is of mutual benefit to researchers in sub-Saharan Africa and Europe. However, a point of concern is that a large percentage of the sub-Saharan African research output results from collaborative projects, meaning that there is still very limited output that comes from purely sub-Saharan African partnerships and our analysis of South-South collaboration links supports this finding. This is in part due to a lack of critical mass of researchers in sub-Saharan Africa, and where local scientific leadership exists, this is mainly established through external research funding that dictates the collaborating partners. This underpins the need to bolster funding for health research and development by African governments as major contributors and to improve policymakers’ understanding of the value of research to drive national health priority setting [16], [17]. Clearly, more political commitment and increased capacity building is needed across the board to enable existing and new research communities in sub-Saharan Africa to not only sustain the current research output but to promote increased research output from South-South collaborations on diseases and needs relevant to the specific regions.

We observed an increasing trend for research output to be published in open-access journals for 2007–2011. Since the early 1990s, scientific peer-reviewed publication has been revolutionised with an increasing number of open-access only journals and, more recently, traditional subscription journals offering an open-access option (hybrid journals) [18], [19]. In addition, many major research funders such as the European Union, Medical Research Council (MRC), Wellcome Trust and the National Institutes of Health (NIH) now require open-access publication of results from the projects that they fund, either in an open-access journal (gold open-access) or in a full-text archives, such as the National Library of Medicine’s PMC (green open-access) [20–23]. According to the European Commission: “open access to scientific peer-reviewed publications has been anchored as an underlying principle of the Horizon 2020 [the European Union Framework programme for Research and Innovation] Regulation and Rules of Participation” [23], [24].

Although, there have been some doubts about the quality of peer-review in some open-access journals, a recent study found that among newer journals, open-access journals were being cited as often as subscription-based journals and, in some subcategories, they are being cited more [18], [25]. Even though some studies have reported no evidence of a citation advantage for open-access journals compared with subscription journals, publishing research results in open-access journals should increase its accessibility to more readers, which is important in sub-Saharan Africa [26], [27].

We used the Web of Science database to analyse the research output on PRDs from European EDCTP member countries and sub-Saharan African countries. This database contains over 12,000 of the highest impact journals, including open-access journals, selected using rigorous editorial and quality criteria. This database has several advantages over others, as it provides data on both scientific productivity and impact through the citation count and includes information on all institutions participating in Thomson’s Reuters Web of Science and their country of origin, which is not included in *Medline*, allowing for the quantification of collaboration between countries that publish these research activities. However, while coverage of English-language journals is very comprehensive, one limitation of the Web of Science is that coverage of non-English-language journals is less extensive, although this has recently increased with the inclusion of French and Portuguese journals in particular. Another potential limitation of this analysis is the choice of the Web of Science as our only data source. This database was the first to be established, but other databases are now available, such as Scopus, which includes nearly 22,000 journals [28]. One study that compared the Web of Science and Scopus reported a higher citation rate for several tropical diseases (including malaria and some NIDs) with Scopus, a significantly higher citation rate for TB with the Web of Science and no significant difference for HIV/AIDS [29]. The author suggested that this was because Scopus abstracts more from biomedical journals than the Web of Science and journals from developing countries are more likely to be included in Scopus. However, the authors did not provide information on how the search strategy was optimised for both databases nor if the results were normalised and both of these factors can have an important impact on the reliability of the results.

A general limitation of our analysis is that bibliometrics is not an ‘exact science’ and relies upon interpretation and re-iteration to achieve a ‘best fit’ dataset that will adequately describe the research area whilst excluding papers of marginal relevance. The results must be interpreted with this in mind. Another potential limitation of our analysis is the method used to assign papers to organisations. Authors often report their affiliations in different ways for different publications, so we used an algorithm to unify these affiliations. International governmental and non-governmental organisations, including organisations such as WHO were assigned to the country given by the author. This may have resulted in an inflated research output for some countries (e.g. Switzerland for WHO, Luxembourg for Médecins sans Frontières). Additionally, we were looking at collaboration patterns based on papers only, whereas some research collaboration takes time to set up and may not produce papers for many years. Given that there is a time lag between research funding and publication and between publication and citation more recently funded research is less likely to be published and less likely to be cited compared with research that has had more time to accumulate citations. As less than 30% of the EDCTP-funded clinical trials had been completed at the time of this analysis, our results inevitably show an incomplete picture. Nonetheless, the results have already revealed an increase in high impact papers across the three main PRDs. We expect the observed trends to continue as suggested by the number of recent, high impact relevant papers that have been published since 2013 and, therefore, were not included in the current analysis [30], [31]. In the future, with more EDCTP-associated papers published, we hope to establish more exact estimates of the difference in research performance and relevant benchmarks as observed in the Impact Profiles.

In conclusion, the normalised citation impact of research from sub-Saharan-Africa increased substantially from 2003 to 2011; collaborative research had a higher impact and was more highly cited than non-collaborative research and EDCTP-funded research was particularly highly cited and published in high impact journals. Since one of the core elements of EDCTP is to promote collaborative research, the association between highly collaborative research and high citation supports the inference that the partnership plays an important role in promoting sub-Saharan African leadership in PRDs research.

## Supporting Information

S1 Checklist PRISMA checklist. (DOC)

S1 Fig Impact profiles of EDCTP-associated papers in HIV-AIDS, 2003–2011. Percentage of research output (number of publications) in HIV/AIDS in each of the eight categories of relative citation rates (x-axis) for EDCTP-associated papers (red solid line) against the global benchmark (in solid grey line) and benchmark for European collaboration with sub-Saharan Africa (dashed red line). The proportion of uncited papers are on the left of the chart.
(DOCX)

S2 Fig Impact profiles of EDCTP-associated papers in tuberculosis, 2003–2011. Percentage of research output (number of publications) in tuberculosis in each of the eight categories of relative citation rates (x-axis) for EDCTP-associated papers (red solid line) against the global benchmark (in solid grey line) and benchmark for European collaboration with sub-Saharan Africa (dashed red line). The proportion of uncited papers are on the left of the chart.
(DOCX)

S3 Fig Impact profiles of EDCTP-associated papers in malaria, 2003–2011. Percentage of research output (number of publications) in malaria in each of the eight categories of relative citation rates (x-axis) for EDCTP-associated papers (red solid line) against the global benchmark (in solid grey line) and benchmark for European collaboration with sub-Saharan Africa (dashed red line). The proportion of uncited papers are on the left of the chart.
(DOCX)

S1 Diagram Modified PRISMA flowchart. (DOCX)

S1 Dataset Excel sheet including all relevant data underlying the findings. (XLSX)
