# Supplementary figures and images for: Bibliometric Assessment of European and Sub-Saharan African Research Output on Poverty-Related and Neglected Infectious Diseases from 2003 to 2011
Source: PLoS Negl Trop Dis. 2015 Aug 11;9(8):e0003997. doi: 10.1371/journal.pntd.0003997 (PMC4532507; doi:10.1371/journal.pntd.0003997)

**Impact Profiles of EDCTP-associated papers in HIV-AIDS, 2003-2011**


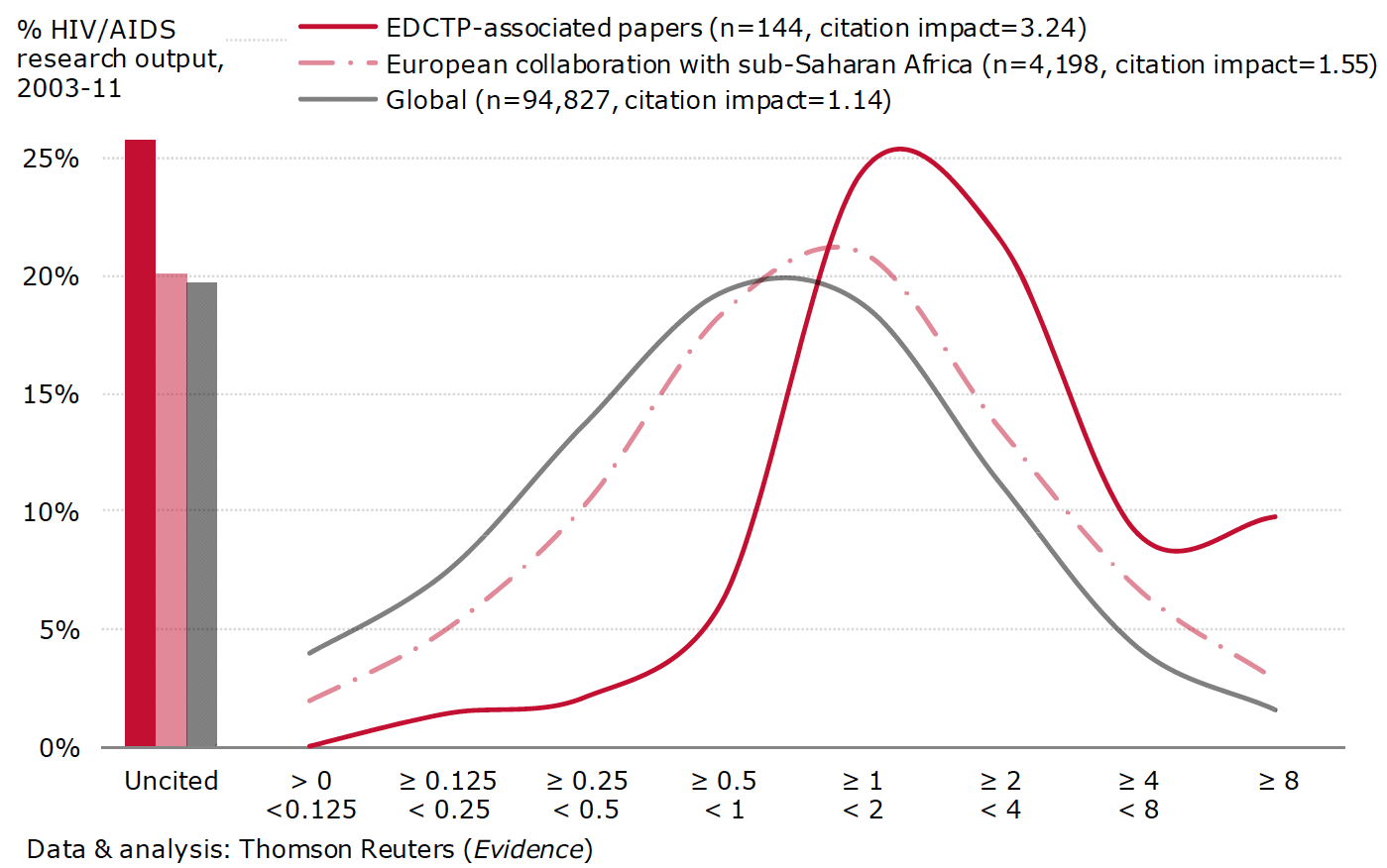

Supplement: S1 Fig — Percentage of research output (number of publications) in HIV/AIDS in each of the eight categories of relative citation rates (x-axis) for EDCTP-associated papers (red solid line) against the global benchmark (in solid grey line) and benchmark for European collaboration with sub-Saharan Africa (dashed red line). The proportion of uncited papers are on the left of the chart. (DOCX) [file pntd.0003997.s002.docx]

**Impact Profiles of EDCTP-associated papers in tuberculosis, 2003-2011**


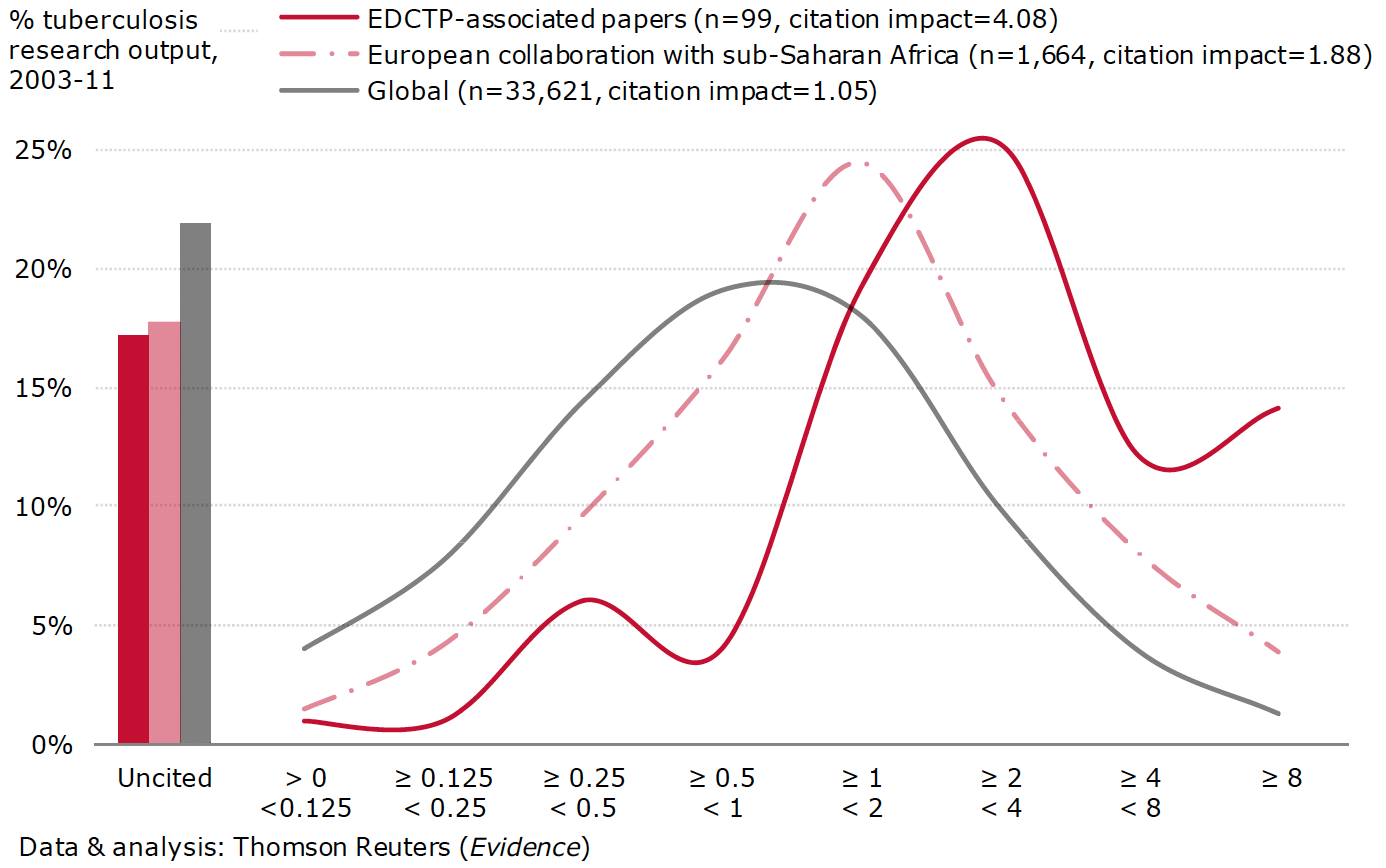

Supplement: S2 Fig — Percentage of research output (number of publications) in tuberculosis in each of the eight categories of relative citation rates (x-axis) for EDCTP-associated papers (red solid line) against the global benchmark (in solid grey line) and benchmark for European collaboration with sub-Saharan Africa (dashed red line). The proportion of uncited papers are on the left of the chart. (DOCX) [file pntd.0003997.s003.docx]

**Impact Profiles of EDCTP-associated papers in malaria, 2003-2011**


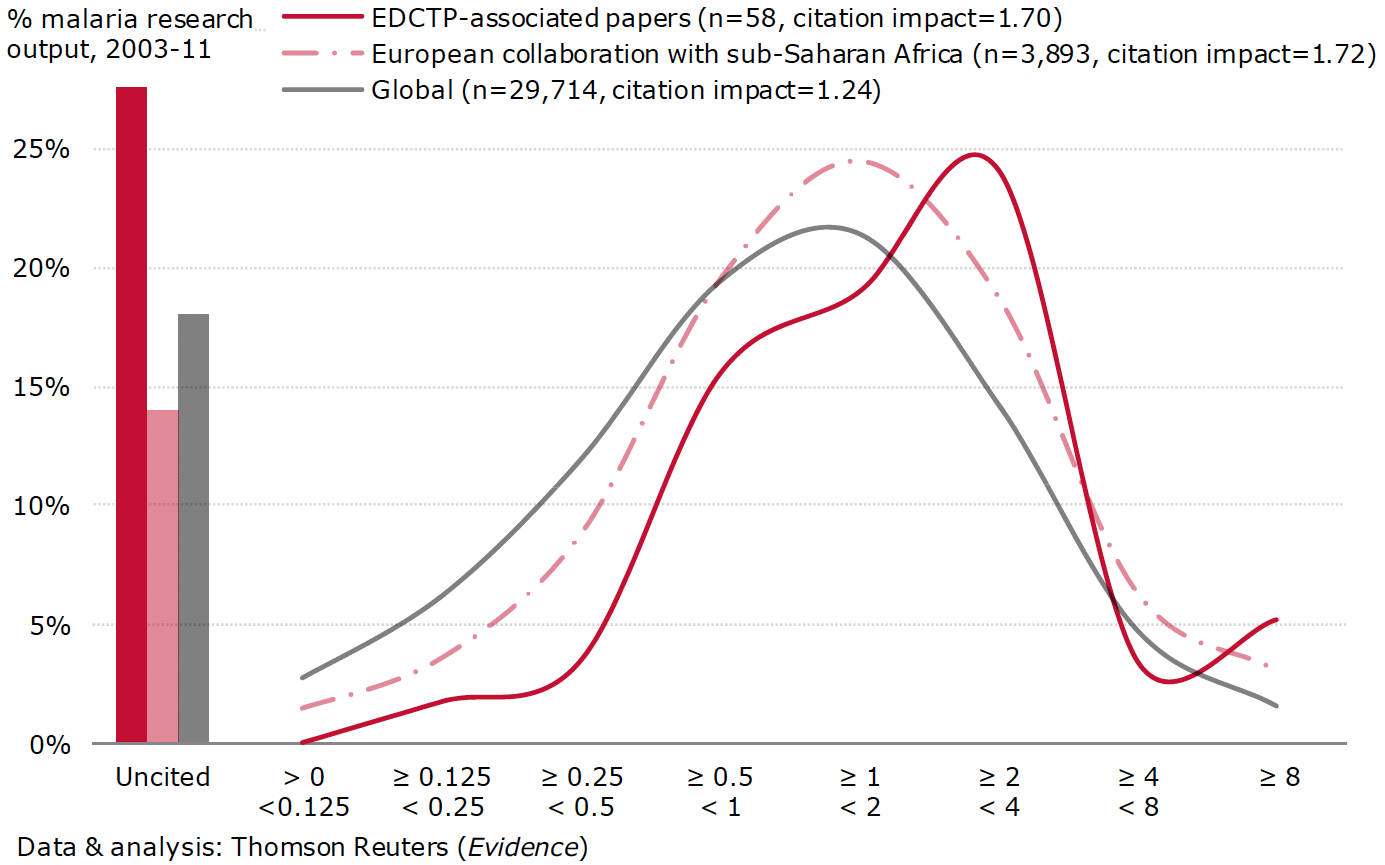

Supplement: S3 Fig — Percentage of research output (number of publications) in malaria in each of the eight categories of relative citation rates (x-axis) for EDCTP-associated papers (red solid line) against the global benchmark (in solid grey line) and benchmark for European collaboration with sub-Saharan Africa (dashed red line). The proportion of uncited papers are on the left of the chart. (DOCX) [file pntd.0003997.s004.docx]
