# Supplementary material for: Bibliometric Assessment of European and Sub-Saharan African Research Output on Poverty-Related and Neglected Infectious Diseases from 2003 to 2011
Source: PLoS Negl Trop Dis. 2015 Aug 11;9(8):e0003997. doi: 10.1371/journal.pntd.0003997 (PMC4532507; doi:10.1371/journal.pntd.0003997)
Supplement: S1 Diagram — (DOCX) [file pntd.0003997.s005.docx]

**Flow diagram for identifying and selecting publications in the field of poverty-related diseases (PRDs) for the bibliometric analysis**

Web of Science

EDCTP in-house publication dataset

(n=244)

EDCTP-associated papers identified in Web of Science (n=437)

EDCTP-associated papers identified in Web of Science dataset 2003-2012 (n=258)

Papers identified through searching Web of Science 2003-2012

(n=290,539)

88,045 papers not included in the citation analysis as citation data only available to end of 2011

Papers remaining after linkage to the Science Citation Index 2003-2011

(n=202,494)

Papers in main dataset used for *disease specific analysis*

(n=202,494)

EDCTP associated papers in sub dataset used for EDCTP *disease specific analysis*

(n=237)

Papers remaining after linkage to PubMed to identify epidemiological and clinical studies

(n=175,900)

Papers in sub dataset used for *study type specific analysis*

(n=175,900)

Note: the boxes in grey represent the different datasets that were used in the various analyses.
